# *Helicobacter pylori* CagA EPIYA Motif Variations Affect Metabolic Activity in B Cells

**DOI:** 10.3390/toxins13090592

**Published:** 2021-08-24

**Authors:** Sebastian Diechler, Bianca E. Chichirau, Gernot Posselt, Dionyssios N. Sgouras, Silja Wessler

**Affiliations:** 1 Division of Microbiology, Department of Biosciences, Paris-Lodron University of Salzburg, 5020 Salzburg, Austria; sebastian.diechler@sbg.ac.at (S.D.); bianca.chichirau@sbg.ac.at (B.E.C.); gernot.posselt@sbg.ac.at (G.P.); 2 Laboratory of Medical Microbiology, Hellenic Pasteur Institute, 127 Vas. Sofias Avenue, 115 21 Athens, Greece; sgouras@pasteur.gr; 3 Cancer Cluster Salzburg, Allergy-Cancer-BioNano Research Centre, Paris-Lodron University of Salzburg, 5020 Salzburg, Austria

**Keywords:** *Helicobacter pylori*, CagA, EPIYA motifs, B cells

## Abstract

Background: *Helicobacter pylori* (*Hp*) colonizes the human stomach and can induce gastric cancer and mucosa-associated lymphoid tissue (MALT) lymphoma. Clinical observations suggest a role for the *Hp* virulence factor cytotoxin-associated gene A (CagA) in pathogenesis. The pathogenic activity of CagA is partly regulated by tyrosine phosphorylation of C-terminal Glu-Pro-Ile-Tyr-Ala (EPIYA) motifs in host cells. However, CagA differs considerably in EPIYA motifs, whose functions have been well characterized in epithelial cells. Since CagA is fragmented in immune cells, different CagA variants may exhibit undetected functions in B cells. Methods: B cells were infected with *Hp* isolates and isogenic mutants expressing different CagA EPIYA variants. CagA translocation and tyrosine phosphorylation were investigated by Western blotting. Apoptosis was analyzed by flow cytometry and metabolic activity was detected by an MTT assay. Results: Isogenic CagA EPIYA variants are equally well translocated into B cells, followed by tyrosine phosphorylation and cleavage. B cell apoptosis was induced in a CagA-independent manner. However, variants containing at least one EPIYA-C motif affected metabolic activity independently of phosphorylation or multiplication of EPIYA-C motifs. Conclusions: The diverse structure of CagA regulates B cell physiology, whereas B cell survival is independent of CagA.

## 1. Introduction

The human pathogen *Helicobacter pylori* (*Hp*) persistently colonizes the epithelial lining of the stomach and causes various gastric disorders including gastritis, ulceration, gastric adenocarcinoma and B cell lymphoma of the mucosa-associated lymphoid tissue (MALT) [1]. In addition to the genetic predisposition of the host, a number of bacterial virulence factors contribute to the pathogenesis of *Hp*. *Hp* cytotoxin-associated gene A (CagA) is an important effector protein that is translocated by the bacterial type IV secretion system (T4SS) into the cytoplasm of infected host cells where it deregulates cancer-associated signal transduction pathways [2]. In fact, epidemiologic data imply a strong association of the development of gastric cancer with the expression of CagA.

Moreover, the formation and progression of MALT lymphomas have been linked to CagA-positive *Hp* strains [3]. The healthy gastric mucosa does not encompass secondary lymphoid organs, whereas *Hp* infections stimulate the formation of MALT in the gastric mucosa, which can develop into MALT lymphoma of B cell origin [4]. Direct contact of *Hp* and B cells is facilitated by the impaired mucosal epithelial barrier, which is mediated by direct *Hp*-mediated opening of intercellular adhesions and chronic inflammation or ulceration during persistent infections, allowing passage of *Hp* to underlying tissues [5,6]. In fact, *Hp* has been reported to be localized in intercellular spaces of epithelial cells, in the lamina propria and in direct contact with infiltrating immune cells in biopsies [7]. Thus, *Hp* can directly interact with B lymphocytes and translocate CagA. Under experimental conditions, *Hp* efficiently delivers CagA into B lymphocytes and activates extracellular signal-regulated kinase 1 and 2 (ERK1/2) and p38 mitogen-activated protein kinase. This, in turn, prevents B cell apoptosis, underlining that CagA may be directly associated with the development of MALT lymphomas [8].

In recent decades, the molecular mechanisms of CagA have been intensively investigated in gastric epithelial cells. Upon CagA translocation into epithelial host cells, intracellular CagA-induced signaling is predominantly controlled by tyrosine phosphorylation sites in the C-terminal EPIYA motifs. Based on the surrounding sequences, the EPIYA motifs can be distinguished as EPIYA-A, -B, -C and -D motifs. Typically, EPIYA-A, -B and variable numbers of -C motifs are found in clinical *Hp* strains isolated from Western populations, whereas East Asian-derived *Hp* strains comprise EPIYA-A, -B and -D motifs [9]. Previous studies have shown that CagA is initially phosphorylated at EPIYA-C motifs by Src kinases in gastric epithelial cells. After early inactivation of Src kinases, c-Abl maintains tyrosine phosphorylation of the EPIYA-C motif and additionally targets EPIYA-A and EPIYA-B motifs [5,10]. A number of phospho- and non-phospho-CagA-dependent cancer-associated signal transduction pathways have been described in gastric epithelial cells [9,11], which are primarily regulated through binding to specific signal molecules [12]. Importantly, the amplification of EPIYA motifs can potentiate CagA-dependent signaling in gastric epithelial cells and the risk of gastric cancer [13,14].

*Hp* delivers CagA into the cytoplasm of infected B cells [8,15,16], where it is then cleaved into a 100 kDa N-terminal and 35 kDa C-terminal fragment, the latter also being phosphorylated at the EPIYA motifs [15,16]. In comparison to epithelial cells, the activation kinetics of the CagA kinases Src and Abl significantly differ in B cells, where they are activated simultaneously [16], indicating a specific function of CagA in B cells. However, the effect of different EPIYA motif variants on CagA phosphorylation and their impact on B cell physiology remains unknown. Hence, a detailed analysis of translocated CagA proteins exhibiting different types and numbers of EPIYA motifs in *Hp*-infected B cells was performed. Clinical isolates and isogenic mutants were used to pinpoint the effect of EPIYA motif variations. It was found that the presence of an EPIYA-C motif plays a role in the metabolic activity of B cells independently of phosphorylation or EPIYA-C amplification.

## 2. Results

**Figure 1:**
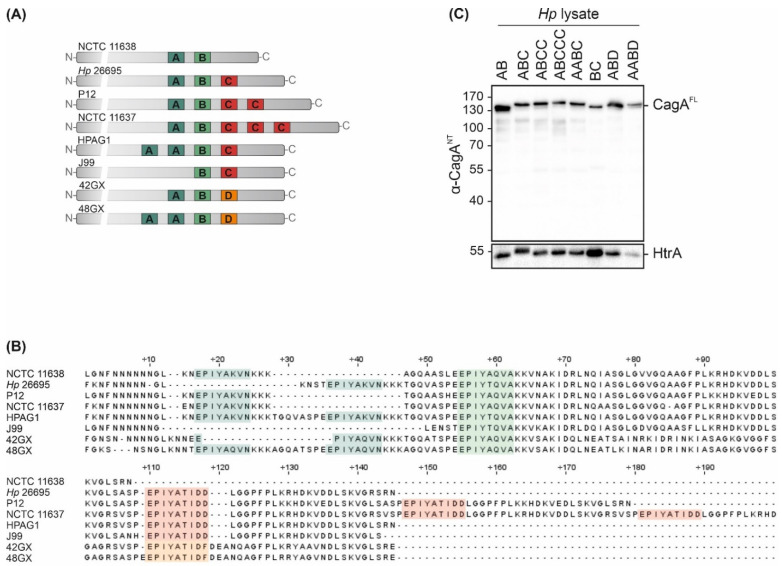
CagA expression in *Hp* isolates. (**A**,**B**) Variable 3‣ terminal regions of *cagA* genes from *Hp* isolates NCTC 11638 (AB), Hp 26695 (ABC), P12 (ABCC), NCTC 11637 (ABCCC), HPAG1 (AABC), J99 (BC), 42GX (ABD) and 48GX (AABD) were sequenced to verify EPIYA-A, -B and -C/D motifs. (**C**) CagA expression was confirmed by Western blot analysis of *Hp* lysates. Full-length CagA (CagA^FL^) was detected using an antibody directed against CagA N-terminus (α-CagA^NT^). Bacterial HtrA was detected as a loading control.

CagA is a crucially important virulence factor of *Hp*, and displays high strain-to-strain variability in C-terminally located EPIYA motifs. To examine the effects of different EPIYA motifs on CagA translocation and phosphorylation, a set of clinical *Hp* isolates of Western origin harboring natural variations of these motifs was investigated. *Hp cagA* genes were sequenced and the expression of EPIYA-AB in NCTC 11638, EPIYA-ABC in *Hp* 26695, EPIYA-ABCC in P12, EPIYA-ABCCC in NCTC 11637, EPIYA-AABC in HPAG1 and EPIYA-BC in J99 was verified (Figure 1A,B). In addition, the *cagA* genes of two East Asian *Hp* strains were analyzed [17], which have not yet been sequenced. Here, an EPIYA-ABD could be detected in strain 42GX and an EPIYA-AABD in 48GX (Figure 1A,B). Western blot analysis of bacterial lysates using an antibody directed against the CagA N-terminus (α-CagA^NT^) revealed the expression of full-length CagA (CagA^FL^) in all tested isolates, while the constitutively *Hp*-expressed serine protease high temperature requirement A (HtrA) was detected as a loading control (Figure 1C).

**Figure 2:**
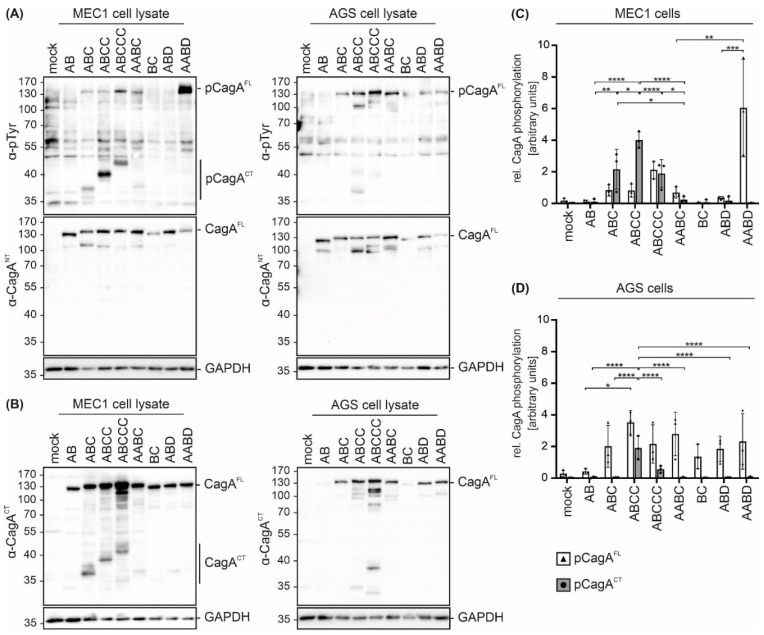
*Hp* isolates translocate CagA harboring different EPIYA motifs into B cells and gastric epithelial cells. (**A**) CagA translocation and phosphorylation in B cell line MEC1 (left panel) and gastric epithelial AGS cells (right panel) were investigated in infection experiments using the indicated *Hp* strains. Cells were infected at an MOI of 100 for 4 h or remained uninfected (mock). Phosphorylated full-length CagA (pCagA^FL^) and C-terminal CagA (pCagA^CT^) were analyzed by Western blotting of whole cell lysates using an anti-phosphotyrosine-specific antibody (α-pTyr). Full-length CagA (CagA^FL^) was detected by α-CagA^NT^. GAPDH was detected as a loading control. (**B**) CagA cleavage was analyzed after infection of MEC1 (left panel) and AGS cells (right panel). CagA was detected using an antibody (α-CagA^CT^) recognizing C-terminal CagA (CagA^CT^) and CagA^FL^. Equal sample loading was confirmed by detection of GAPDH. (**C**,**D**) pCagA^FL^ (white bars) and pCagA^CT^ (grey bars) were quantified in MEC1 (**C**) and AGS cells (**D**) by blot densitometry, normalized to the loading control GAPDH and expressed as arbitrary units (* *p* < 0.05, ** *p* < 0.01, *** *p* < 0.001, **** *p* < 0.0001).

To analyze the effects of different EPIYA motifs on CagA phosphorylation in B cells, the B cell chronic lymphocytic leukemia (B-CLL) cell line MEC1 was utilized as an established infection model [15,16]. MEC1 cells were infected with all tested strains and cell lysates were analyzed by Western blotting. Translocation and phosphorylation of full length CagA (pCagA^FL^) and C-terminal CagA fragments (pCagA^CT^) were detected (Figure 2A, left panel) using an anti-phosphotyrosine (α-pTyr) antibody. *Hp* CagA^FL^ and GAPDH were shown as controls. pCagA^CT^ and/or pCagA^FL^ were observed in infections with *Hp* isolates containing EPIYA-ABC, EPIYA-ABCC, EPIYA-ABCCC, EPIYA-AABC and EPIYA-AABD motifs, but not in CagA proteins harboring EPIYA-AB, EPIYA-BC, or EPIYA-ABD (Figure 2A, left panel), which likely reflect individual differences in *Hp* strains. Similar results were obtained in *Hp*-infected gastric epithelial AGS cells (Figure 2A, right panel). In line with a previous study [10], pCagA^FL^ was detectable when at least one EPIYA-C or -D motif was present. Accordingly, an increasing intensity of pCagA^FL^ corresponding to the number of EPIYA-C motifs was observed. As reported previously, pCagA^FL^ signals for J99 were weak [10]. After infection with EPIYA-ABCC- and EPIYA-ABCCC-expressing *Hp* strains, a weak signal for a tyrosine-phosphorylated ~40 kDa protein was also detected in AGS cells (Figure 2A, right panel), which could be a putative phosphorylated CagA fragment as previously described [18].

CagA cleavage in both cell lines was verified by detection of CagA^FL^ and C-terminal cleavage fragments (CagA^CT^) using an antibody (α-CagA^CT^) that was raised against the C-terminus (Figure 2B). In contrast to the low amounts of cleaved EPIYA-AB, EPIYA-AABC, EPIYA-BC, EPIYA-ABD and EPIYA-AABD-containing CagA proteins, cleavage of EPIYA-ABC, EPIYA-ABCC and EPIYA-ABCCC was evident in MEC1 cells, albeit to different extents (Figure 2B, left panel). In gastric epithelial cells, CagA fragmentation has not been described as a frequent or particularly strong process, but the consistent appearance of an N-terminal CagA protein fragment exhibiting a size of approximately 100 kDa (Figure 2A, right panel) and low amounts of C-terminal fragments (Figure 2B, right panel) support a weak cleavage or breakage of CagA. Hence, it was assumed that CagA fragments also appear in epithelial cells and are detected with higher sensitivity using anti-phosphotyrosine or anti-CagA^CT^ antibodies when the CagA protein contains more EPIYA motifs. The quantification of phosphorylated CagA in MEC1 (Figure 2C) or AGS cells (Figure 2D) demonstrated different pCagA^FL^ to pCagA^CT^ ratios in the two cell lines, which indicates (i) varying phosphorylation and cleavage properties in individual strains and (ii) strong CagA fragmentation in MEC1 cells (Figure 2C). Altogether, these data also support the finding that EPIYA-C motifs are the primary phosphorylation targets not only in gastric epithelial cells [18], but also in B cells, despite the described early activation of c-Abl kinases [16].

**Figure 3:**
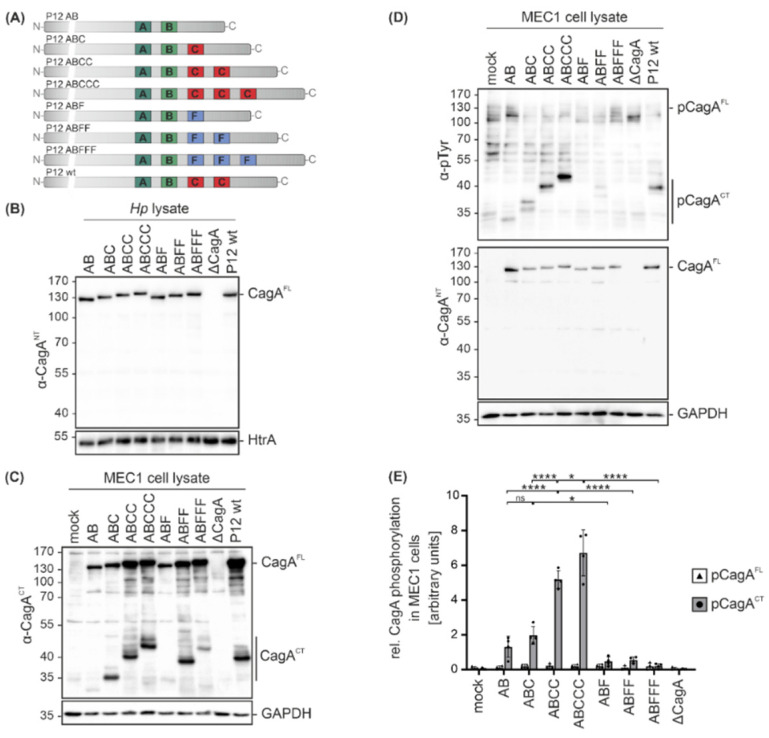
Infections with isogenic *Hp* mutant strains reveal that the presence of an EPIYA-C motif enhances CagA phosphorylation. (**A**) The variable 3´ terminal regions of *cagA* genes containing EPIYA-C motifs (AB, ABC, ABCC, ABCCC) or phosphorylation-deficient EPIFA motifs (ABF, ABFF, ABFFF) of isogenic *Hp* P12 mutants and P12 wild types (wt) were verified by sequencing. (**B**) Expression of CagA variants in bacterial lysates was analyzed by Western blotting. Full-length CagA (CagA^FL^) was detected using a CagA N-terminal antibody (α-CagA^NT^). Detection of HtrA served as a loading control. (**C**) MEC1 cells were infected at an MOI of 100 for 4 h with the indicated *Hp* mutants, P12 wt, or remained uninfected (mock). CagA cleavage in MEC1 cells was analyzed using an anti-CagA antibody (α-CagA^CT^) recognizing the C-terminal region of CagA (CagA^CT^) and CagA^FL^. GAPDH was detected as a loading control. (**D**) Phosphorylation of full-length CagA (pCagA^FL^) and C-terminal CagA fragments (pCagA^CT^) was analyzed using antibodies against phosphorylated tyrosine (α-p-Tyr) and CagA^FL^ was detected by α-CagA^NT^. (**E**) pCagA^FL^ (white bars) and pCagA^CT^ (grey bars) were quantified by blot densitometry, normalized to GAPDH and expressed as arbitrary units (* *p* < 0.05, **** *p* < 0.0001, ns: not significant).

Since clinical *Hp* isolates not only express different CagA variants, but also display different genetic backgrounds, isogenic *Hp cagA* mutants in *Hp* strain P12 were analyzed [13] to investigate the functional consequences of CagA EPIYA motif variations. In addition to single EPIYA-A and -B motifs (AB), CagA proteins contain defined numbers of EPIYA-C motifs (ABC, ABCC, ABCCC) or the respective phosphorylation-deficient EPIFA-C motifs (ABF, ABFF, ABFFF) [13] (Figure 3A). The expression levels of CagA were compared in bacterial lysates from P12 *cagA* mutants, a P12 *cagA* knock-out mutant (Δ*cagA*) and P12 wild type (wt) by Western blot analysis using the α-CagA^NT^ antibody, which confirmed equal CagA expression in all strains. HtrA was detected as a loading control (Figure 3B). MEC1 cells were then infected with the indicated *Hp cagA* mutants followed by Western blot analysis. To identify C-terminal CagA cleavage fragments (CagA^CT^), an antibody directed against the C-terminal CagA region was used and GAPDH expression was analyzed to show equal protein loading (Figure 3C). Cleavage was detectable for all CagA variants with a stronger signal for EPIYA-ABC, EPIYA-ABCC, EPIYA-ABCCC, EPIYA-ABFF and EPIYA-ABFFF (Figure 3C). Whether the EPIYA-AB and EPIYA-ABF are cleaved to a lesser extent or the corresponding C-terminal fragments are weakly recognized by the anti-CagA^CT^ antibody needs to be further investigated in the future. CagA translocation and phosphorylation were investigated utilizing the α-pTyr or α-CagA^NT^ antibodies (Figure 3D). pCagA^FL^ and pCagA^CT^ were quantified from four independent experiments (Figure 3E). Phosphorylation of C-terminal CagA fragments (pCagA^CT^) gradually increased in direct proportion to the number of EPIYA-C motifs, whereas the EPIYA-AB variant showed strongly reduced phosphorylation (Figure 3D,E). As previously described, phosphorylation of CagA^FL^ in B cells was undetectable [15,16], indicating immediate cleavage of CagA after translocation (Figure 3C,D). The CagA proteins containing phosphorylation-deficient EPIYA-ABF, EPIYA-ABFF and EPIYA-ABFFF were not tyrosine-phosphorylated (Figure 3D,E), underlining the importance of EPIYA-C motifs in the hierarchical cascade of CagA phosphorylation.

**Figure 4:**
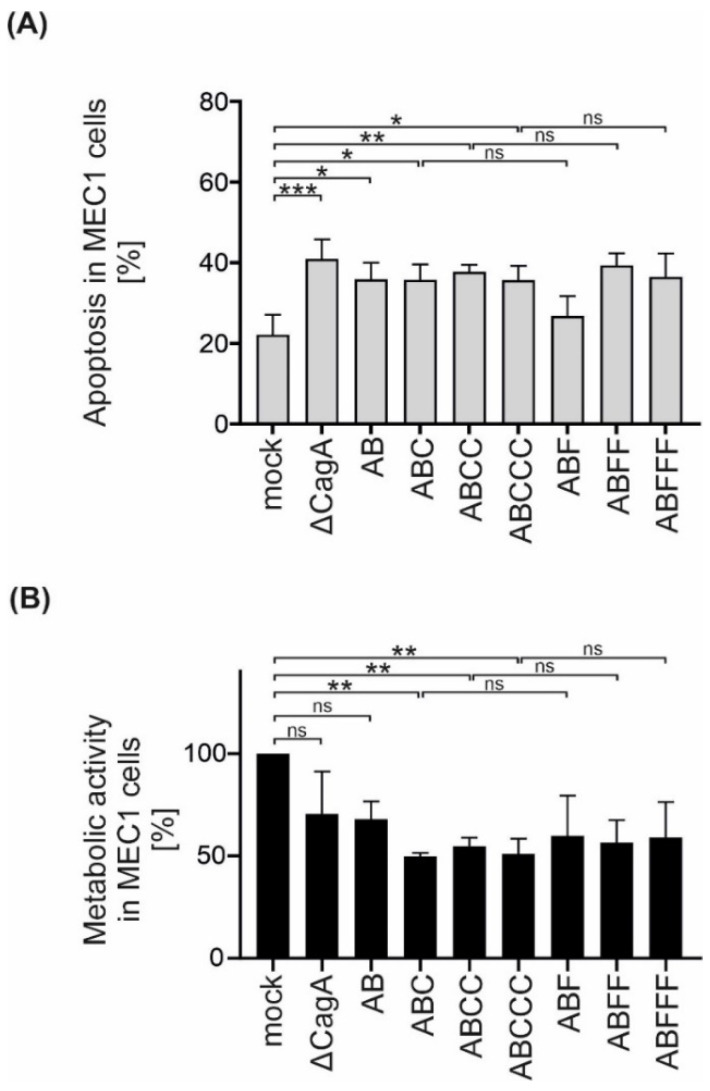
Isogenic *Hp* mutants induce apoptosis in B cells independently of CagA, but decrease metabolic activity dependently on EPIYA-C motifs. (**A**) MEC1 cells were infected with P12 mutants at an MOI of 50 as indicated. Apoptosis was measured after 24 h by flow cytometry. Results represent the means of three independent experiments (* *p* < 0.05, ** *p* < 0.01, *** *p* < 0.001, ns: not significant). (**B**) MEC1 cells were infected with *Hp* strains at an MOI of 200. After 24 h, metabolic activity was measured using an MTT assay. The data represent the results from three independent experiments, each performed in triplicate. The metabolic activities of *Hp*-infected cells are shown in relation to the mock control (** *p* < 0.01, ns: not significant).

CagA has been shown to affect apoptotic signaling in B lymphocytes [8]. Since EPIYA-C motif repetitions increased CagA phosphorylation in B cells, the effect of EPIYA-C motifs on the survival and metabolic activity of MEC1 cells was investigated. Therefore, apoptosis of B cells was analyzed and it was observed that all *Hp* strains increased apoptosis independently of CagA (Figure 4A). However, the metabolic activity, which is mainly mediated through NAD(P)H-dependent cellular reductases, was decreased in infections with P12 Δ*cagA* and to a similar extent in P12 EPIYA-AB compared to uninfected MEC1 cells (Figure 4B). Interestingly, the presence of a single EPIYA-C or phosphorylation-deficient EPIFA-C motif contributed to a further significant decrease in metabolic activity compared to P12 Δ*cagA* and P12 EPIYA-AB. This effect was not further strengthened by additional EPIYA-C or EPIFA-C motifs present in EPIY/FA-ABCC and EPIY/FA-ABCCC. Based on these observations, the effect of the EPIYA-C motif on metabolic activity proved to be independent of tyrosine phosphorylation (Figure 4B).

## 3. Discussion

CagA is a polymorphic virulence factor with high genetic variability and is strongly associated with the outcome of gastric disorders in response to persistent *Hp* infections. The variability of CagA is characterized by different signatures and numbers of EPIYA motifs harboring tyrosine phosphorylation sites that are important for CagA functions. In gastric epithelial cells, kinases from the Src and Abl families were identified to phosphorylate CagA in a time- and motif-dependent manner [10,19]. The amino acid sequences surrounding the EPIYA motifs determine the binding specificity of different EPIYA motifs to distinct signaling molecules, which control cellular responses such as proliferation, inflammation, cell morphology, or migration [12].

*Hp* efficiently translocates CagA into several types of immune cell, where it is tyrosine-phosphorylated and cleaved into a 100 kDa N-terminal and a tyrosine-phosphorylated 35 kDa C-terminal fragment [20,21]. In AGS cells, CagA fragmentation can be observed as well [18]. However, compared to MEC1 cells, the ratio of pCagA^FL^ to pCagA^CT^ fragments differs significantly in epithelial cells, suggesting that CagA fragmentation is less prominent, follows different kinetics, or that fragments have a shorter half-life. However, the function of CagA and its dependence on the EPIYA motif configuration in B cells is poorly understood. In this study, the translocation, phosphorylation and cleavage of different natural and engineered *Hp* CagA variants were investigated using the MEC1 cell line as an infection model for B cells [15,16]. It was found that clinical *Hp* isolates expressing different CagA types translocated CagA into B cells, followed by tyrosine phosphorylation and cleavage. Importantly, in our previous study, it was shown that CagA cleavage in MEC1 cells is independent of tyrosine phosphorylation [16]. CagA phosphorylation is also mediated by Src and Abl kinases in MEC1 cells, which, however, are activated simultaneously [16], while a consecutive activation is observed in epithelial cells [10,19]. Whether these kinases also exhibit preferences for individual EPIYA motifs in B cells was not analyzed in this study.

In gastric epithelial cells, phosphorylation of the EPIYA-C motif was identified as a crucially important step, which primes phosphorylation of EPIYA-A or EPIYA-B motifs. Furthermore, it has been demonstrated that in CagA EPIYA-ABC or CagA EPIYA-ABD proteins, a maximum of two EPIYA motifs are phosphorylated simultaneously [10]. However, it remains unknown whether triple phosphorylation occurs at CagA molecules harboring multiple EPIYA-C motifs. In this report, phosphorylation of CagA EPIYA-AB in AGS and MEC1 was weak, also confirming the importance of the EPIYA-C motif in priming phosphorylation of EPIYA-A and -B motifs in MEC1 cells, despite the early activation of Abl family kinases [16]. The addition of one EPIYA-C motif induced stronger phosphorylation of CagA^CT^, which was further enhanced by multiple copies of the EPIYA-C motif, suggesting that repetitions of the EPIYA-C motif might allow triple or quadruple phosphorylation of CagA.

In our previous reports, it was found that inhibition of Src and Abl kinases decreased B cell death, implying the direct involvement of tyrosine kinases or pCagA in cell viability [15,16]. Previous work by Lin and colleagues showed that CagA interacts with SHP-2 phosphatase to activate ERK1/2 (extracellular signal-regulated kinase 1 and 2) and p38 kinases. Together with the upregulation of anti-apoptotic proteins Bcl-2 and Bcl-X, it was suggested that translocated CagA can promote B cell survival [8]. Here, infection of B cells with P12 *Hp* mutants resulted in apoptosis independently of CagA and EPIYA motifs. It was observed that the metabolic activity of B cells was strongly decreased by *Hp* expressing CagA EPIYA-AB type, which was amplified by CagA proteins harboring at least one EPIYA-C motif. Additional EPIYA-C motif copies did not contribute a further reduction in metabolic activity, which is in line with *Hp*-induced interleukin-8 (IL-8) expression and secretion from AGS cells, which is independent of EPIYA-C repeats as well [22]. Interestingly, the decrease in metabolic activity was independent of EPIYA-C motif phosphorylation. These data suggest that CagA EPIYA-ABC proteins have an impact on the metabolic activity of B cells separate from affecting apoptosis or survival.

## 4. Conclusions

It has been shown that CagA proteins containing at least one EPIYA-C motif target B cell physiology during infection, which could diminish anti-*Hp* antibody responses and foster persistent colonization of the host [23]. In conclusion, these data indicate that CagA has an impact on the fate of B cells during *Hp* infection.

## 5. Materials and Methods

### 5.1. Bacteria

*Hp* isolates NCTC 11638 (DSMZ), *Hp* 26695 [24], P12 [25], NCTC 11637 (DSMZ), HPAG1 [26], J99 [27], 42GX [17] and 48GX [17] were cultivated on agar plates supplemented with 10% horse serum as previously described [19]. The generation of isogenic P12 mutants has been described previously [13]. Bacteria were cultivated at 37 °C under microaerobic conditions using CampyGen Oxoid bags (Thermo Fisher Scientific, Austria) for 24 h. All *Hp cagA* sequences were validated by sequencing.

### 5.2. Sequencing

Genomic DNA (gDNA) was extracted from *Hp* with phenol-chloroform-isoamylalcohol (pH 8.0). The gDNA was precipitated in 100% ethanol and 0.3 M NaOAc (pH 5.2) supplemented with linear polyacrylamide (Sigma-Aldrich, Taufkirchen, Germany) at 4 °C. For PCR assays, two universal primers for amplification and sequencing of C-terminal *cagA* were designed (5′-GAT TTC AGC AAG GCA GAA GAA AC-3′ (forward) and 5′-CGC TTC CCA CAT TAT GCG CAA C-3′ (reverse)). C-terminal *cagA* sequences were amplified in 20 µL PCR reactions using DreamTaq polymerase (Thermo Fisher, Austria) and 30 ng of gDNA as a template. PCR amplicons were separated on 1% agarose gels and fragments were eluted with GeneJET gel extraction kits (Thermo Fisher, Vienna, Austria). Sequencing of PCR fragments was performed by Eurofins genomics (MWG, Munich, Germany).

### 5.3. Infection Experiments

AGS (ECACC, 89090402) and MEC1 (DSMZ, ACC-497) cells were cultivated in RPMI 1640 containing 1% L-glutamine and 10% fetal calf serum (FCS). AGS cells were cultivated in 10 cm dishes for 48 h to reach a density of 5 × 10^6^ cells per dish. Prior to infection, the medium was aspirated and AGS cells were cultivated in starving medium (RPMI 1640, 1% L-glutamine) overnight. MEC1 cells were seeded 16 h before infection in starving medium at a density of 5 × 10^6^ cells per dish. For infection experiments, the bacteria were resuspended in PBS and the concentration was determined by optical density measurements at 600 nm. AGS and MEC1 cells were treated with *Hp* at a multiplicity of infection (MOI) of 100 for indicated time periods. Equal volumes of PBS were added for mock controls. After 4 h of infection, images were taken using a phase-contrast microscope (CKX41, Olympus). Cells were washed with ice-cold PBS and lysed in a modified RIPA buffer (20 mM Tris pH 7.5, 1 mM EDTA, 100 mM NaCl, 0.5% DOC, 0.1% SDS, 1% Triton x-100, 20 mM β-glycerophosphate, 20 mM NaF, 1 mM Na_2_MoO_4_, 1 mM Na_3_VO_4_, 1 × complete protease inhibitor cocktail (Roche Diagnostics)).

### 5.4. Immunoblotting

Fifty micrograms of whole cell lysates were loaded on 8–10% polyacrylamide gels and separated by SDS PAGE. Proteins were transferred onto nitrocellulose or PVDF membranes (Carl Roth, Karlsruhe, Germany) using semidry transfer blotting. Membranes were blocked in 3% bovine serum albumin (BSA) supplemented with 0.05 mM Na_3_VO_4_ and incubated with primary antibodies overnight at 4 °C. CagA was analyzed using polyclonal anti-CagA antibodies detecting the N-terminal region of CagA (Paul-Ehrlich Institute, Langen, Germany) [16] or the C-terminal region (raised against amino acids 890-1186) [15]. Phosphorylated CagA was detected using the anti-phosphotyrosine antibody 4G10 (gift from Prof. Stephan Feller, Halle an der Saale, Germany), anti-GAPDH antibody recognized GAPDH (Cell Signaling, Frankfurt am Main, Germany) and anti-HtrA antibody raised against *Hp* HtrA (Paul-Ehrlich Institute, Langen, Germany) [28]. Densitometric analysis of Western blots was performed from at least three to four independent experiments. For quantification, band intensities were measured with Image Lab software (version 6.0.1, Bio-Rad, Vienna, Austria) and normalized to GAPDH. Statistics were analyzed with one-way ANOVA followed by Tukey’s test for multiple comparison correction.

### 5.5. Apoptosis Assays and Metabolic Activity

MEC1 cells were serum-starved and seeded in 6-well plates 1 h before infection. Briefly, cells were infected with *Hp* strains at an MOI of 50 for 24 h. Cells were analyzed according to the manufacturer’s instructions with an FITC Annexin V apoptosis detection kit (BD Biosciences, Vienna, Austria). Apoptosis was calculated from three independent experiments by analyzing the combined FITC Annexin V/propidium-iodide double-positive and FITC Annexin V single-positive cells. Metabolic activity was analyzed using an MTT assay according to the manufacturer’s instructions (Sigma-Aldrich, Darmstadt, Germany). Cells were infected in 96-well plates for 24 h at an MOI of 200. After incubation with 3-(4,5-dimethyl-2-thiazolyl)-2,5-diphenyl-2H-tetrazolium bromide (MTT) for 1 h, cells were lysed in 0.1% NP-40, 40 mM HCl in 2-propanol and metabolic activity was measured at 570 nm using an Infinite 200 Pro reader (Tecan, Grödig, Austria). Reference measurements were performed at 630 nm. Metabolic activity was calculated from three independent experiments in triplicate and measurements were normalized to respective mock controls. For apoptosis and MTT assays, normal distribution was analyzed using the Shapiro–Wilk test and statistics were calculated using one-way ANOVA. Tukey’s test was used for multiple comparison correction.

## Data Availability

Data are contained within the article.
